# Impact of Remote Consultations on Antibiotic Prescribing in Primary Health Care: Systematic Review

**DOI:** 10.2196/23482

**Published:** 2020-11-09

**Authors:** Seung Min Han, Geva Greenfield, Azeem Majeed, Benedict Hayhoe

**Affiliations:** 1 School of Public Health Imperial College London London United Kingdom; 2 Department of Primary Care and Public Health School of Public Health Imperial College London London United Kingdom

**Keywords:** remote consultations, antibiotic, primary health care, systematic review, consultation, telehealth, COVID-19, safety, prescription

## Abstract

**Background:**

There has been growing international interest in performing remote consultations in primary care, particularly amidst the current COVID-19 pandemic. Despite this, the evidence surrounding the safety of remote consultations is inconclusive. The appropriateness of antibiotic prescribing in remote consultations is an important aspect of patient safety that needs to be addressed.

**Objective:**

This study aimed to summarize evidence on the impact of remote consultation in primary care with regard to antibiotic prescribing.

**Methods:**

Searches were conducted in MEDLINE, Embase, HMIC, PsycINFO, and CINAHL for literature published since the databases’ inception to February 2020. Peer-reviewed studies conducted in primary health care settings were included. All remote consultation types were considered, and studies were required to report any quantitative measure of antibiotic prescribing to be included in this systematic review. Studies were excluded if there were no comparison groups (face-to-face consultations).

**Results:**

In total, 12 studies were identified. Of these, 4 studies reported higher antibiotic-prescribing rates, 5 studies reported lower antibiotic-prescribing rates, and 3 studies reported similar antibiotic-prescribing rates in remote consultations compared with face-to-face consultations. Guideline-concordant prescribing was not significantly different between remote and face-to-face consultations for patients with sinusitis, but conflicting results were found for patients with acute respiratory infections. Mixed evidence was found for follow-up visit rates after remote and face-to-face consultations.

**Conclusions:**

There is insufficient evidence to confidently conclude that remote consulting has a significant impact on antibiotic prescribing in primary care. However, studies indicating higher prescribing rates in remote consultations than in face-to-face consultations are a concern. Further, well-conducted studies are needed to inform safe and appropriate implementation of remote consulting to ensure that there is no unintended impact on antimicrobial resistance.

## Introduction

Recent years have seen unsustainable workload increases in primary health care. Remote consultations, in which primary care professionals (PCPs) communicate with patients by telephone or internet as an alternative to face-to-face consultations, have been implemented to maximize the efficiency of primary care services and meet patient demand for greater and more convenient access to primary health care advice [1]. Over 90 countries have been reported to be already delivering health care services over the telephone in 2016 [1], and remote consultations have been playing a substantial role in the health care response to the current COVID-19 pandemic by supporting continued access to services with minimized risk of disease transmission [2,3].

The increasingly commonplace nature of remote consulting in primary care notwithstanding, there remains much uncertainty regarding the safety and effectiveness of remote consultations [4,5]. For example, PCPs are more likely to prescribe medications in remote consultations than in face-to-face settings [6,7]. In particular, antibiotic prescribing behavior can be influenced by nonclinical factors that are unique to remote consultations, such as the inability to physically examine the patient [8]. The overprescribing of antibiotics drives antimicrobial resistance, which is a global concern with consequent impact on patients and health systems, especially primary health care. Over 80% of all antibiotic prescriptions are dispensed by primary care in the United Kingdom [9-11].

Evidence for the impact of remote consultations on antibiotic prescribing in primary care is currently unclear. Given the growing international adoption of remote consultations into primary care, which has intensified during the ongoing COVID-19 pandemic, any impact of remote consultations on antibiotic prescribing needs to be properly understood. This study aims to summarize the impact of remote consultations on primary care antibiotic prescribing.

## Methods

This systematic review was conducted following the PRISMA (Preferred Reporting Items for Systematic Reviews and Meta-Analyses) guidelines (Multimedia Appendix 1) [12].

### Eligibility Criteria

Included studies were required to be conducted in primary health care settings only. Any type of remote consultations between patient and PCP were accepted as the intervention. For comparison, any form of face-to-face consultation in primary health care was accepted. The primary outcomes of interest were quantitative measures of antibiotic prescriptions in remote consultations. The proportion of guideline-concordant antibiotic prescriptions and follow-up visit rates were considered secondary outcomes. Studies assessing the prescribing rates of any drug without providing data on antibiotics were excluded.

We included peer-reviewed primary research articles written in English in this systematic review. Studies could be observational or randomized controlled trials (RCTs), but conference abstracts, editorials, and qualitative studies that did not provide measures of antibiotic prescribing frequency were excluded. We did not exclude literature based on publication date so that we could capture all available literature.

### Search Strategy

A scoping review was conducted to establish the search terms. A research librarian was consulted for guidance regarding the search strategy. An initial list of search terms was developed and applied to MEDLINE and Embase to check the relevance of results, and reference lists from several relevant studies and similar reviews were manually searched to expand the search terms. Search strings were then amended according to the subject headings for each database. The final list of search terms for each database is presented in Multimedia Appendix 2.

### Study Selection

A search was conducted on February 14, 2020, in 5 electronic databases: MEDLINE, Embase, PsycINFO, HMIC, and CINAHL. We had 3 reviewers independently screen studies for inclusion. One reviewer (SH) screened all titles and abstracts, and BH and GG each screened 50% of the titles and abstracts. The same approach was subsequently performed for full-text screening. Discrepancies were resolved through discussion between the reviewers. The studies were stored using Mendeley reference management software, and duplicates were removed through Mendeley’s deduplication function and manual searching.

### Data Extraction

Study characteristics and outcomes were extracted using a Microsoft Excel spreadsheet. The data extraction form was created in advance and finalized after piloting it on two studies. Data from included studies were extracted by SH.

### Risk of Bias

The National Heart, Lung, and Blood Institute tool [13] was used to assess observational studies, and the Cochrane risk of bias tool [14] was applied to RCTs. The reviewers carefully considered the efforts required to minimize the risk of bias for each domain and ensured that the overall quality rating of each paper was not purely based on the tally of each appraisal form. SH assessed the quality of all papers and BH and GG each assessed half of the studies independently. Conflicting assessments and overall risk of bias were determined through discussion.

### Analysis

Results were presented as reported by the original authors of each study, and similar outcomes that were reported frequently were grouped together for analysis. The results were presented through narrative synthesis [15]. The included studies were considered too heterogeneous in terms of study population, type of consultation, and outcome definitions for meta-analysis.

## Results

### Study Selection

**Figure 1:**
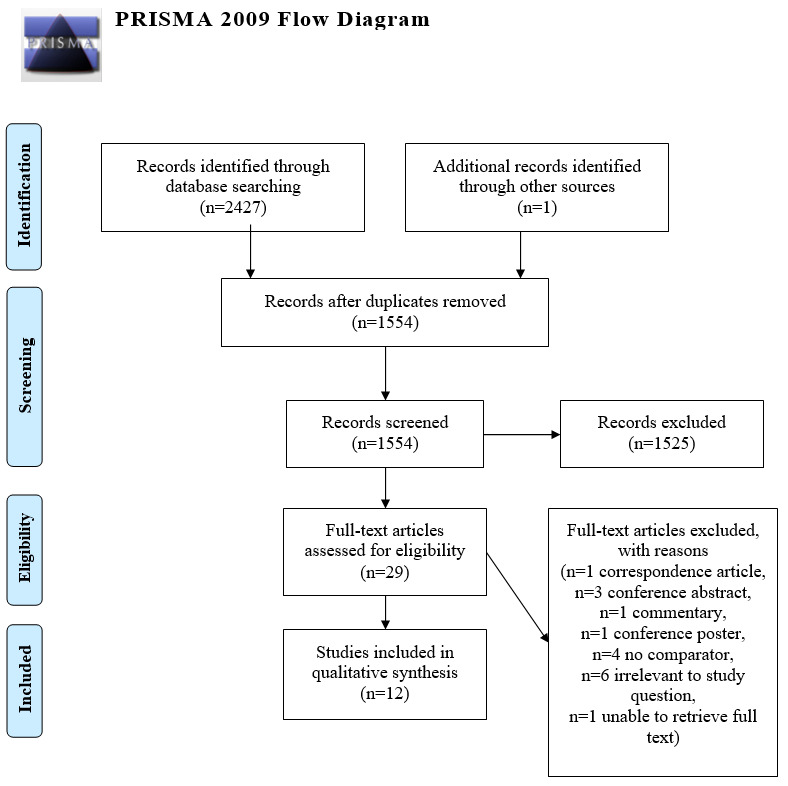
Literature search flow chart adapted from PRISMA (Preferred Reporting Items for Systematic Reviews and Meta-Analyses) flow chart.

Our electronic database search yielded 2427 results, of which 12 studies were included in this review (Figure 1) [12]. We found 2 papers [16,17] that were part of the same study with identical study periods and participants, so the results most relevant to this systematic review were reported.

### Characteristics of Included Studies

The characteristics and results of the 12 studies are summarized in Tables 1 and 2 [16-27]. Of these 12 studies, 9 were conducted in the United States [18,19,22-27], 2 were conducted in Denmark [16,17], and 1 was conducted in Norway [20]. Most studies (11/12) [16-20,22-27] had a cohort design, including 1 RCT [21].

**Table 1:** Summary of study characteristics.

Study (author, year)	Country	Setting	Study design	Population	Remote consultation type
Rokstad and Straand, 1997 [20]	Norway	Primary care practices	Prospective cohort	All registered patients	Telephone, letter, or through a messenger (mixed)
McKinstry et al, 2002 [21]	Scotland	Primary care practices	Randomized controlled trial	All patients that phoned for a same-day appointment	Telephone
Mehrotra et al, 2012 [22]	United States	Primary care practices	Retrospective cohort	All patients	Text-based e-visit
Huibers et al^a^, 2014 [16]	Denmark	Out-of-hours primary care contacts	Retrospective cohort	All registered patients	Telephone
Ewen et al, 2015 [23]	United States	Primary care practices	Retrospective cohort	All registered patients	Telephone
Uscher-Pines et al, 2016 [24]	United States^b^	Primary care practices	Retrospective cohort	Adults aged 18-64 years	Telephone, video, internet, or mobile app consultation (mixed)
Christensen et al^a^, 2016 [17]	Denmark	Out-of-hours primary care contacts	Retrospective cohort	All registered patients	Telephone
Shi et al, 2018 [25]	United States^b^	Primary care practices	Retrospective cohort	Adults aged 18-64 years	Audio and audio-visual conferencing (mixed)
Ray et al, 2019 [27]	United States^b^	Primary care practices	Retrospective cohort	Children aged 0-17 years	Audio-only or audio-video conferencing (mixed)
Murray et al, 2019 [18]	United States	Primary care practices or retail clinics	Retrospective cohort	Women aged 18-65 years	Telephone and text-based e-visit
Johnson et al, 2019 [26]	United States	Primary care practices	Retrospective cohort	Adults aged ≥18 years	Text-based e-visit
Penza et al, 2020 [19]	United States	Primary care retail clinics	Retrospective cohort	Children aged 18 months-18 years	Telephone and text-based e-visit

^a^Articles published on the same study.

^b^The data were sourced from national health insurance companies. Therefore, no specific setting was recorded.

**Table 2:** Main findings of studies.

Indication for antibiotics, study (author, year)			Remote consultations, N	Face-to-face consultations (control), N	Outcome measures	Results
**Antibiotic-prescribing rate higher in remote consultations**						
	**Mehrotra et al, 2012 [22]**					
		Sinusitis	475	4690	Antibiotic-prescribing rate^a^	Intervention: 99%, control: 94% (*P*<.001)
		Urinary tract infection	99	2855	Antibiotic-prescribing rate^a^	Intervention: 99%, control: 49% (*P*<.001)
	**Uscher-Pines et al, 2016 [24]**					
		Uncomplicated acute bronchitis	168	7342	Antibiotic-avoidance rate^b^	Intervention: 16.7%, control: 27.9% (*P*<.01)
	**Ray et al, 2019 [27]**					
		Acute respiratory infection	4604	38408	Antibiotic-prescribing rate^a^	Intervention: 52%, control: 31% (*P*<.001)
	**Penza et al^c^, 2020 [19]**					
		Conjunctivitis	101	202	Antibiotic-prescribing rate^a^ during telephone consultations	Intervention: 41.6%, control: 19.8% (*P*<.0001)
**Antibiotic-prescribing rate lower in remote consultations**						
	**Rokstad and Straand, 1997 [20]**					
		Not specified	24983	42202	Proportion of prescriptions and antibiotics prescriptions made through each consultation type	Antibiotic-prescribing rate^a^ lower in intervention* Intervention: 43.5% of all prescriptions; 7.8% of remote prescriptions were antibiotics Control: 56.6% of all prescriptions; 17.8% of face-to-face prescriptions were antibiotics
	**Huibers et al^d^, 2014 [16]**					
		Not specified	382748	180032	Antibiotic-prescribing rate^a^	Intervention: 26.1% (95% CI 25.9-26.3) Control: 10.7% (95% CI 10.6-10.8)
	**Ewen et al, 2015 [23]**					
		Not specified	61707^e^	61707^e^	Proportion of antibiotics prescriptions out of all prescriptions	12.4% of all antibiotics prescriptions made through telephone (6617 telephone consultations and 27,487 office consultations; 63,418 antibiotics were prescribed during 61,707 consultations to 31,302 individuals)*
	**Shi et al, 2018 [25]**					
		Acute respiratory infection	38839	942163	Antibiotic-prescribing rate^a^	Intervention: 52%, control: 53% (*P*<.01)
	**Johnson et al, 2019 [26]**					
		Sinusitis	175	175	Antibiotic-prescribing rate^a^	Intervention: 68.6%, control: 94.3% (*P*<.001)
**No significant difference in antibiotic-prescribing rate**						
	**McKinstry et al, 2002** **[21]**					
		Not specified	187	181	Antibiotic-prescribing rate^a^	Intervention: 19.3%, control: 16.0%; difference: −3.3% (95% CI −11.1% to 4.5%)
	**Murray et al, 2019 [18]**					
		Urinary tract infection	150	150	Antibiotic-prescribing rate^a^ in telephone consultations	Intervention: 81%, control: 83% (*P*=.76)
		Urinary tract infection	150	150	Antibiotic-prescribing rate^a^ from text-based e-visits	Intervention: 81%, control: 83% (*P*=.65)
	**Penza et al^c^, 2020 [19]**					
		Conjunctivitis	101	202	Antibiotic-prescribing rate^a^ from text-based e-visits	Intervention: 25.7%, control: 19.8% (*P*=.24)

^a^Antibiotic-prescribing rate: percentage of consultations that resulted in antibiotic management per consultation type.

^b^Antibiotic-avoidance rate: percentage of patients that did not receive antibiotics for uncomplicated acute bronchitis, as they had no clinical indication.

^c^Results of the same study for different populations reported separately.

^d^[16] and [17] are articles published on the same study. The results from the Huibers et al [16] study are reported in this table.

^e^Number of remote consultations and face-to-face consultations in this study was not available. The number of consultations altogether has been reported instead.

*P values or confidence intervals not reported in the original studies.

The study population varied in age and sex depending on whether the study investigated specific conditions. Of 2 studies analyzing antibiotic prescribing for urinary tract infection (UTI) [18,22], 1 limited their participants to adult women [18]. Additionally, 2 studies focused only on children with conjunctivitis or acute respiratory infection [19,27].

A majority of studies (7/12) employed telephone consultations, text-based e-visits, or both as their intervention [17-19,21-23,26]. Moreover, 2 studies [25,27] grouped audio-only and video consultations for their intervention, and another study [20] grouped all consultations made through telephone, letters, or messengers. The control arm was face-to-face primary care clinic consultations for all except 2 studies. The Penza et al [19] control group was made up of walk-in retail clinic patients, and the controls for Murray et al [18] were retail clinic and primary care practice patients. Furthermore, while most consultations were evaluated by primary care physicians, telephone consultations in the Penza et al [19] and Murray et al [18] studies were evaluated by registered nurses. Text-based e-visits were assessed by advanced practice providers in the Murray et al [18] study and nurse practitioners in the Penza et al [19] study.

### Risk of Bias Within Studies

The quality assessment is presented in Multimedia Appendix 3. The quality of studies was generally fair, with 7 moderate-quality studies, including the RCT, and 5 observational studies that were considered high quality. All studies had clearly defined their objectives, study population, and exposure and outcome measures. However, only 2 studies provided sample size justifications or power calculations [17,26]. Additionally, 4 studies lacked adjustment for any confounding factors [16,18,20,23]. The RCT described adequate randomization, but lacked blinding [21]. However, we did not consider this to significantly impact the quality, since blinding to consultation type is rarely feasible.

### Impact of Remote Consultations on Antibiotic Prescribing Behavior

The main findings are outlined in Table 2, with confidence intervals and *P* values reported where available. In most studies, the impact of remote consultation on antibiotic prescribing was measured by the percentage of consultations with one or more antibiotic prescriptions for each type of consultation. This measure has been uniformly referred to as the antibiotic-prescribing rate in this review, following the practice in the included studies.

We found 3 studies that reported the antibiotic-prescribing rate to be higher in remote settings than face-to-face consultations [19,22,27]. The difference in prescribing rate ranged from 5% (n=201) to 50% (n=5165) [22]. Penza et al found this relationship for both telephone and text-based e-visits, but only the difference in telephone consultations was statistically significant [19]. Another study concluded that fewer antibiotics were avoided for bronchitis patients consulting over the phone; patients were more likely to receive an antibiotic for the same condition when consulting remotely [24].

We also found 3 cohort studies that reported patients were more likely to be prescribed an antibiotic through face-to-face consultations [16,25,26]. The differences in prescribing rates were generally smaller in these studies; 1 study found a 1% difference (n=1,336,867) [25], and the largest difference observed was 25.7% (n=350) [26]. Additionally, 2 moderate-quality studies, an RCT and a retrospective cohort study, noted no significant difference in prescribing rate between the different settings [18,21].

It was difficult to compare 2 studies with the other included studies due to differences in outcome measures; neither included a direct estimate of antibiotic prescribing. Rokstad and Straand [20] and Ewen et al [23] claimed that antibiotic-prescribing rate was higher in face-to-face consultations based on the percentage of antibiotic prescriptions issued through each consultation type. However, their results do not consider multiple prescriptions made in one consultation. Therefore, their claims should be considered with caution.

Clinicians may be more likely to prescribe antibiotics for UTI in remote consultations. Of 2 studies assessing patients with suspected or confirmed UTI, 1 study found higher antibiotic-prescribing rates in remote consultations [22]. Mehrotra et al [22] concluded that PCPs were less likely to order a UTI-relevant test in face-to-face consultations (did not order: 8%, did order: 51%; P<.01), but more likely to prescribe an antibiotic for UTI in remote consultations. Another moderate-quality study found no significant difference in antibiotic-prescribing rate for patients with UTI [18]. However, the researchers hypothesized that their results could have been affected by differences in face-to-face settings; face-to-face consultations in the Mehrotra et al [22] study were performed in retail clinics where there may have been less continuity of care than primary care practices.

Results for respiratory infections were mixed. In 3 studies, of which 2 were of high quality, researchers found higher antibiotic-prescribing rates in remote consultations than in direct PCP consultations [22,24,27]. However, 2 other high-quality studies noted lower antibiotic-prescribing rates for patients consulting remotely [25,26].

### Impact of Remote Consultations on Guideline-Concordant Prescribing Rate

We found 4 observational studies that reported guideline-concordant prescribing rates or guideline-recommended prescribing rates, which measured the appropriateness of the prescriptions against local or national guidelines [22,25-27] (Table 2). All studies were from the United States and investigated populations with a confirmed diagnosis of sinusitis [22,26], UTI [22], or acute respiratory infection [25,27].

In contrast to the findings for antibiotic-prescribing rate, the guideline-concordant antibiotic management for sinusitis and patients with UTI revealed no significant difference between remote and face-to-face consultations [22,26]. However, conflicting results were observed for patients with acute respiratory infection [25,27].

### Impact of Remote Consultations on Follow-Up Visits

We found 5 US-based retrospective cohort studies that investigated follow-up visit rates after initial consultation for the same presentation [18,19,22,25,26]. Of these 5 studies, 3 found that patients who were seen remotely were more likely to have another follow-up visit than those who attended face-to-face consultations [19,25,26]. Shi et al [25] found this to be true for those who followed up within 2-21 days after their first visit. Results from the Penza et al [19] study indicated higher follow-up rates in both e-visits and telephone consultations than in face-to-face consultations 7 days after the initial visit. Johnson et al [26] found this relationship to be true for text-based e-visits relating to sinusitis in the subsequent 24 hours and 30 days after the initial consultation, but found no difference in follow-up consultations at 7 days after the initial consultation. However, Murray et al [18] and Mehrotra et al [22] found no significant difference in follow-up visit rates between the consultation types within the following 3 weeks and 30 days after the initial consultation, respectively. Further, Murray et al [18] found no significant difference in antibiotic-prescribing rates in the initial consultation for patients who were followed up with.

## Discussion

### Principal Results

To our knowledge, this is the first systematic review to examine how antibiotic prescribing is affected by remote consultation in primary care. This review of moderate- to high-quality studies found that evidence regarding the impact of remote consultations on antibiotic prescribing was mixed. Studies reporting higher antibiotic-prescribing rates in remote consultations than in face-to-face consultations were generally of better quality. However, the inconsistency of results and the small number of studies make it difficult to draw strong conclusions for the effect of remote consultations on antibiotic prescribing.

The studies examining specific indications for antibiotics suggested that antibiotic-prescribing rates for patients with UTI in remote consultations was higher than in face-to-face consultations, but 1 study did not find any difference [18]. Guideline-concordant prescribing rates for patients with UTI or sinusitis were not significantly changed by remote consultations. However, there was mixed evidence regarding whether remote consultations were more likely to be followed up with another consultation for the same condition.

### Limitations

We tried to conduct a comprehensive search by manually searching reference lists to find relevant search terms for remote consultation. However, there is significant variation in terminology among researchers, and this could have led to the omission of a few relevant papers. Other challenges we faced while conducting this review included the dearth of relevant papers. The included studies were conducted in 1 of 4 high-income countries. Given the growing attention on remote consultations in low- and middle-income countries [1], it is possible that relevant papers in grey literature or papers written in languages other than English exist, but were excluded due to our selection criteria. Additionally, the effect of the setting of studies is unaccounted for. For example, retail clinics differ from primary care practices, as there is less chance of establishing a long-term doctor-patient relationship. Moreover, in some studies, remote consultations were evaluated by nurse practitioners, who may have had a different skillset compared to primary care physicians who consulted with patients face-to-face. With the small number of studies, no clear pattern emerged in terms of the impact of setting on the outcome.

### Comparison With Prior Work

Compared to face-to-face consultations, PCPs order fewer tests and investigations and are unable to physically examine patients in remote settings, which can affect the appropriateness of patient management [8,28]. Clinicians consulting in retail clinics via private telemedicine providers could feel pressured to prescribe antibiotics due to the expectations of patients who pay to see clinicians and the diagnostic uncertainty that stems from the lack of continuity in these commercial remote consultations [29]. On the other hand, Banks et al [30] noted that many remote consultations were followed up with face-to-face appointments for adequate clinical assessment. As antibiotic prescriptions made in follow-up appointments contribute to prescriptions made in face-to-face consultations, this could provide a partial explanation for why some of the included studies found lower antibiotic-prescribing rates in initial remote consultations and higher frequencies of follow-up consultations [25,26].

However, research on the remote prescribing of antibiotics is too limited to make useful comparisons. A systematic review of reviews on the benefits of telemedicine in 2002 found too little high-quality evidence to confidently conclude that telemedicine was beneficial. However, the review reported that research was beginning to address the literature gaps in the field [31]. More recent reviews agree that there is a need for more research on the safety of care [4] and the quality and safety of prescribing through remote consultations [4,32]. As this is a novel study, it is difficult to compare the findings of this review directly with other reviews. We hope that this review serves as a reference point for future studies.

### Implication for Research and Practice

We anticipate that remote consultations will continue to be used frequently following the COVID-19 pandemic, leading to a different mix of cases involving remote consultations compared to the mix of cases seen prior to the pandemic. Future studies should focus on conducting trials that adjust for this difference in case makeup. Moreover, the current literature is reliant on studies from high-income countries and observational studies, as confirmed by the inclusion of only one RCT in this review. Randomized trials from a variety of geographical settings are needed to achieve balance in the literature.

Antibiotic prescribing should only occur when it is safe, clinically indicated, and likely to be of benefit, regardless of consultation type [33]. Consequently, the quality and safety of antibiotic prescribing in remote consultations should be comparable to that of face-to-face consultations. Similar guideline-concordant prescribing between remote and face-to-face consultations is reassuring, but the evidence for whether the management at remote consultations is effective is less clear. Follow-up visits after remote consultations are often necessary per clinicians’ advice and patients’ need for physical examinations. Therefore, a high follow-up visit rate does not necessarily correlate with poor management at initial consultation, but it does raise the question of the effectiveness (including cost-effectiveness) of remote consultations. Future research investigating the resolution of symptoms as an endpoint could be beneficial.

The divided weight of evidence in this review makes it difficult to inform health policy, as the evidence regarding remote consultations is still evolving. Nonetheless, good quality evidence suggesting higher antibiotic-prescribing rate in remote consultations should not be ignored. As remote consultations are being used more frequently due to the pandemic, clear guidelines and criteria for face-to-face consultations and antibiotic management are needed. Meanwhile, PCPs should continue to be cautious when prescribing antibiotics and remain attentive to local and national guidelines. Furthermore, current antibiotic stewardship programs [34,35] could be adapted and implemented into remote care to encourage appropriate prescribing.

### Conclusions

We found inconsistent evidence across the included studies for the impact of remote consulting on antibiotic prescribing in primary care. However, as the use of remote consultations continues to increase in primary care worldwide, ensuring the safety and quality of these consultations, including avoiding adverse impacts on antimicrobial resistance, should be prioritized. Studies indicating higher prescribing rates than face-to-face consulting are a concern, and PCPs should be cautious when considering prescribing antibiotics through remote consultations*.* Randomized trials are needed in a variety of geographical settings to inform policy on the wide-scale implementation of remote consultations. This type of research should be a priority, as long-term increases in remote consulting in primary care seems to be an inevitable consequence of the global COVID-19 pandemic.

### Disclaimer

This paper presents independent research commissioned by the National Institute for Health Research under the Applied Health Research Programme for North West London. The views expressed in this publication are those of the author(s) and not necessarily those of the National Health Service, National Institute for Health Research, or Department of Health.

## Appendix

Multimedia Appendix 1 Checklist of items to include when reporting a systematic review or meta-analysis.

Multimedia Appendix 2 Search terms and strategy for each electronic database.

Multimedia Appendix 3 Quality assessment for observational studies and randomized trials.

## Abbreviations

PCP: primary care professionals
RCT: randomized controlled trial
UTI: urinary tract infection
