# Supplementary material for: Impact of Remote Consultations on Antibiotic Prescribing in Primary Health Care: Systematic Review
Source: J Med Internet Res. 2020 Nov 9;22(11):e23482. doi: 10.2196/23482 (PMC7655728; doi:10.2196/23482)
Supplement: Multimedia Appendix 2 [file jmir_v22i11e23482_app2.pdf]

MEDLINE (through OVID)

#1<br>Antibiotics	anti-bacterial agents/ *or* antibiotic* *or* anti-biotic* *or* antibacterial* *or* anti-bacterial* *or* antimicrobial* *or* anti-microbial* *or* antiinfective* *or* anti-infective*
#2<br>Remote consultation	remote consultation/ *or* telemedicine/ or exp teleconsultation/ or exp telehealth/ or tele* or mobile applications/ or mobile app* or remote* or skype or phone* or video* or web* or internet* or email* or real-time or realtime or online or virtual* or e-consult* or econsult* or electronic consult* or electronic communicat* or ecommunicat* or e-communicat* or eadvi#e or e-advi#e or electronic advi#e or e-health or ehealth or e-visit* or evisit*
#3<br>Primary care	Exp general practice/ *or* family practice/ *or* exp general practitioners/ *or* primary health care/ *or* physicians, family/ *or* physicians, primary care/ *or (*primary adj2 care) *or* primary healthcare *or* general practice* or family practice* or general practitioner* *or* general physician* *or* GP *or* family adj2 care *or* family doctor* *or* family medic* *or* family physician* *or* ambulatory care

Search strategy: #1 AND #2 AND #3

EMBASE (through OVID)

#1<br>Antibiotics	*antibiotic agent/ *or* antibiotic* *or* anti-biotic* *or* antibacterial* *or* anti-bacterial* *or* antimicrobial* *or* anti-microbial* *or* antiinfective* *or* anti-infective*
#2<br>Remote consultation	Telemedicine/ or exp teleconsultation/ or exp telehealth/ or tele* or mobile application/ or mobile health application/ *or* mobile app* or remote* or skype or phone* or video* or web* or internet* or email* or real-time or realtime or online or virtual* or e-consult* or econsult* or electronic consult* or electronic communicat* or ecommunicat* or e-communicat* or eadvi#e or e-advi#e or electronic advi#e or e-health or ehealth or e-visit* or evisit*
#3<br>Primary care	Exp general practice/ *or* exp general practitioner/ *or* exp primary medical care/ *or* primary adj2 care *or* primary healthcare *or* general practice* *or* family practice* *or* general practitioner* *or* general physician* *or* GP *or* family physician* *or* family medic* *or* ambulatory care *or* exp primary health care/ *or* exp primary medical care/ *or* family adj2 care *or* family doctor*

Search strategy: #1 AND #2 AND #3

HMIC (through OVID)

#1 Antibiotics	exp antibiotics/ *or* antibiotic* *or* anti-biotic* *or* antibacterial* *or* anti-bacterial* *or* antimicrobial* *or* anti-microbial* *or* antiinfective* *or* anti-infective*
#2 Remote consultation	Exp telehealth/ or exp telemedicine/ or e*xp* telecare/ *or* exp telephone consultations/ or tele* or mobile app* or remote* or skype or phone* or telephone* or video* or web* or internet* or email* or real-time or realtime or online or virtual* or e-consult* or econsult* or electronic consult* or electronic communicat* or ecommunicat* or e-communicat* or eadvi#e or e-advi#e or electronic advi#e or e-health or ehealth or e-visit* or evisit*
#3 Primary care	Exp general practice/ *or* exp general practitioners/ *or* exp primary care/ *or* primary adj2 care *or* primary healthcare *or* general practice* *or* family practice* *or* general practitioner* *or* general physician* *or* GP *or* family physician* *or* family medicine *or* ambulatory care *or* family adj2 care *or* family doctor* *or* family medic* practitioner*

Search strategy: #1 AND #2 AND #3

PSYCINFO (through OVID)

#1 Antibiotics	exp Antibiotics/ *or* antibiotic* *or* anti-biotic* *or* antibacterial* *or* anti-bacterial* *or* antimicrobial* *or* anti-microbial* *or* antiinfective* *or* anti-infective*
#2 Remote consultation	Telemedicine/ or teleconsultation/ or exp Mobile Applications/ or tele* or mobile app* or remote* or skype or phone* or video* or web* or internet* or email* or real-time or realtime or online or virtual* or e-consult* or econsult* or electronic consult* or electronic communicat* or ecommunicat* or e-communicat* or eadvi#e or e-advi#e or electronic advi#e or e-health or ehealth or e-visit* or evisit*
#3 Primary care	exp General Practitioners/ *or P*rimary Health Care/ or Family Medicine/ or Family Physicians/ *or* primary adj2 care *or* primary healthcare *or* general practice* *or* family practice* *or* general practitioner* *or* general physician* *or* GP *or* family physician* *or* family medic* *or* ambulatory care *or* family adj2 care *or* family doctor*

Search strategy: #1 AND #2 AND #3

CINAHL (through EBSCOhost)

#1<br>Antibiotics	(MH “Antibiotics”) *OR* antibiotic* *OR* anti-biotic* OR antibacterial* OR anti-bacterial* OR antimicrobial* OR anti-microbial* OR antiinfective* OR anti-infective*
#2<br>Remote consultation	(MH “Remote Consultation”) OR (MH “Telemedicine”) OR (MH “Telehealth”) OR (MH “Mobile Applications”) OR tele* OR mobile app* OR remote* OR skype OR phone* OR video* OR web* OR internet* OR email* OR real-time OR realtime OR online OR virtual* OR e-consult* OR econsult* OR electronic consult* OR electronic communicat* OR ecommunicat* OR e-communicat* OR eadvi#e OR e-advi#e OR electronic advi#e OR e-health OR ehealth OR e-visit* OR evisit*
#3<br>Primary care	(MH “Physicians, Family”) OR (MH “Family Practice”) OR (MH “Primary Health Care”) OR primary N1 care OR primary healthcare OR general practice* OR family practice* OR general practitioner* OR general physician* OR GP OR family physician* OR family medic* OR ambulatory care OR family N1 care OR family doctor*

Search strategy: #1 AND #2 AND #3
