# Supplementary material for: Impact of Remote Consultations on Antibiotic Prescribing in Primary Health Care: Systematic Review
Source: J Med Internet Res. 2020 Nov 9;22(11):e23482. doi: 10.2196/23482 (PMC7655728; doi:10.2196/23482)
Supplement: Multimedia Appendix 3 [file jmir_v22i11e23482_app3.pdf]

Quality assessment of observational studies (NHLBI Tool for Observational Cohort and Cross-sectional Studies

Author	Q1	Q2	Q3	Q4	Q5	Q6	Q7	Q8	Q9	Q10	Q11	Q12	Q13	Q14	Overall
Rokstad	Y	Y	Y	Y	N	N	Y	NA	Y	N	Y	N	NA	N	Mod
Mehrotra	Y	Y	NA	Y	N	Y	Y	NA	Y	N	Y	NA	NR	N	Mod
Huibers	Y	Y	NR	Y	N	Y	Y	NA	Y	NA	Y	NA	NR	N	Mod
Ewen	Y	Y	NR	Y	N	Y	Y	NA	Y	NA	N	CD	NR	N	Mod
Uscher-Pines	Y	Y	Y	Y	N	N	Y	NA	Y	N	Y	N	NR	Y	High
Christensen	Y	Y	NR	Y	Y	Y	Y	NA	Y	NA	Y	NA	NR	Y	Mod
Shi	Y	Y	Y	Y	N	N	Y	NA	Y	NA	Y	N	NR	Y	High
Ray	Y	Y	Y	Y	N	Y	Y	NA	Y	N	Y	N	NR	Y	High
Murray	Y	Y	NA	Y	N	Y	Y	NA	Y	NA	Y	NA	NR	N	Mod
Johnson	Y	Y	N	Y	Y	Y	Y	NA	Y	NA	Y	NA	NR	Y	High
Penza	Y	Y	NA	Y	N	N	Y	NA	Y	NA	Y	N	NA	Y	High

Q1. Was the research question or objective in this paper clearly stated? **Q2.** Was the study population clearly specified and defined? **Q3.** Was the participation rate of eligible persons at least 50%? **Q4.** Were all the subjects selected or recruited from the same or similar populations (including the same time period)? Were inclusion and exclusion criteria for being in the study prespecified and applied uniformly to all participants? **Q5.** Was a sample size justification, power description, or variance and effect estimates provided? **Q6.** For the analyses in this paper, were the exposure(s) of interest measured prior to the outcome(s) being measured? **Q7.** Was the timeframe sufficient so that one could reasonably expect to see an association between exposure and outcome if it existed? **Q8.** For exposures that can vary in amount or level, did the study examine different levels of the exposure as related to the outcome (e.g., categories of exposure, or exposure measured as continuous variable)? **Q9.** Were the exposure measures (independent variables) clearly defined, valid, reliable, and implemented consistently across all study participants? **Q10.** Was the exposure(s) assessed more than once over time? **Q11.** Were the outcome measures (dependent variables) clearly defined, valid, reliable, and implemented consistently across all study participants? **Q12.** Were the outcome assessors blinded to the exposure status of participants? **Q13.** Was loss to follow-up after baseline 20% or less? **Q14.** Were key potential confounding variables measured and adjusted statistically for their impact on the relationship between exposure(s) and outcome(s)?

Q, question; CD, cannot be determined; NA, not applicable; NR, not reported; N, No; Y, Yes; Mod, Moderate

Quality assessment of randomised trial using the Cochrane Risk of Bias tool

Study	Risk of bias							Overall Quality
	Random sequence generation	Allocation concealment	Blinding of participants & personnel	Blinding of outcome assessment	Incomplete outcome data	Selective reporting	Other sources of bias	
**McKinstry**	L	L	H	H	L	L	H	M

L, low; M, moderate; H, high
